# Scutellarin Alleviates CCl_4_-Induced Liver Fibrosis by Regulating Intestinal Flora and PI3K/AKT Signaling Axis

**DOI:** 10.3390/ijms26072997

**Published:** 2025-03-25

**Authors:** Xin Li, Wanqi Yang, Ying Weng, Yingying Zhao, Haidong Chen, Yang Chen, Jishuang Qiu, Bei Jiang, Chunyan Li, Yong Lai

**Affiliations:** 1 Yunnan Key Laboratory of Screening and Research on Anti-Pathogenic Plant Resources from Western Yunnan (Cultivation), Dali University, Dali 671000, China; 13688795252@163.com (X.L.); jiangbei@dali.edu.cn (B.J.); 2 College of Pharmacy, Dali University, Dali 671000, China; yangwanqi@dali.edu.com (W.Y.); m13108878353@163.com (Y.W.); 17863811495@163.com (Y.Z.); 17748976035@163.com (H.C.); cy18973599761@163.com (Y.C.); qmqm973973@163.com (J.Q.)

**Keywords:** liver fibrosis, gut microbiota, fecal microbiota transplantation, metabolomics, network pharmacology, transcriptomics, PI3K/AKT pathway

## Abstract

Liver fibrosis is a pathological manifestation of chronic liver disease developing to the terminal stage, and there is a lack of effective therapeutic drugs in clinical practice. Scutellarin (SCU) is a flavonoid extracted from *Erigeron breviscapus (Vaniot.) Hand.-Mazz.*, which has significant anti-liver-fibrosis efficacy, but its mode of action remains incompletely understood. A liver fibrosis model was built with male Sprague Dawley rats induced with the disease by CCl_4_ to evaluate the therapeutic effect of drugs. 16S rRNA sequencing and metabolomics were used to analyze the regulatory effects of SCU on intestinal flora and host metabolism; antibiotics were administered to eliminate gut microbiota and fecal microbiota transplantation (FMT) experiments were used to verify the mechanism. The mechanistic basis underlying SCU’s hepatic anti-fibrotic effects was screened by network pharmacology combined with transcriptomics, combined with molecular docking, qPCR, and WB verification. The results showed that SCU may play an anti-liver-fibrosis role by correcting the imbalance of gut flora and regulating the linoleic acid and purine metabolic pathways. In addition, SCU can downregulate the levels of proteins and genes related to the PI3K/AKT axis. In summary, SCU alleviates liver fibrosis by reversing intestinal flora imbalance, regulating the metabolic profile, and inhibiting the PI3K/AKT axis.

## 1. Introduction

Liver fibrosis is a frequent pathological occurrence in numerous chronic liver conditions, and its progression can induce irreversible end-stage cirrhosis, liver failure, and even hepatocellular carcinoma. According to statistics, over 2 million individuals worldwide succumb to liver diseases annually, accounting for 4% of all deaths [1]. Although anti-liver-fibrosis drugs have been clinically utilized, there remain safety concerns such as complex drug mechanisms and large toxic side effects. Consequently, it is critical to explore low-toxicity and effective anti-liver-fibrosis therapeutics [2].

The multi-target and low toxicity characteristics of traditional Chinese medicine provide rich resources for the development of anti-fibrosis drug candidates [3]. Scutellarin (SCU), a flavonoid component derived from the dried whole herb of *Erigeron breviscapus (Vant.) Hand.-Mazz.*, demonstrates multiple biological functions such as antioxidant properties; anti-inflammatory, pain-relieving, and liver-protective effects; etc. [4,5]. The oral administration of SCU and its derivatives has been shown to partially reverse liver fibrosis [6,7]. However, the therapeutic effect and mechanism of SCU in liver fibrosis still need further evaluation.

Interestingly, flavonoids have poor pharmacokinetic characteristics and low bioavailability; they demonstrate significant biological effects and exhibit high pharmacological activity [8]. Based on this, research on the mechanism accounting for flavonoids has centered on gut flora [9]. The presence of the ‘intestinal-liver axis’ results in flora imbalance and intestinal barrier damage during liver fibrosis progression, further worsening liver fibrosis and forming a harmful cycle [10]. In recent years, protecting the liver by improving intestinal flora disorders has gained significant scholarly attention. Empirical evidence suggests demonstrated that supplementing the probiotic *Lactobacillus rhamnosus GG* can inhibit liver damage and fibrosis caused by cholestatic liver disease in mice [11]. Furthermore, increasing evidence suggests that maintaining intestinal homeostasis has potential therapeutic or diagnostic value [12,13]. Therefore, the application of fecal microbiota transplantation (FMT) to reconstitute perturbed microbial ecosystems and return the intestinal microbiota that has been altered by disease-related conditions to a “healthy” balance has become a new scientific disease treatment method. In recent years, the regulation of intestinal flora through fecal microbiota transplantation (FMT) has been included in the treatment of some diseases [14]. This indicates that intestinal flora may serve as an anti-liver-fibrosis target. In addition, intestinal microbial metabolites are also strongly connected to the onset of liver diseases [10,15]. Meanwhile, SCU exhibits crosstalk with gut flora and prevents intestinal microecological imbalance, and its prebiotic activity potentially inhibits the pathogenic-bacteria-induced secretion of inflammatory mediators [6]. However, whether the therapeutic benefits conferred by SCU against liver fibrosis are mediated by intestinal bacteria remains unclear.

Therefore, we hypothesized that SCU may help treat liver fibrosis by regulating the intestinal flora and their anti-inflammatory ability. In this study, we used 16S rRNA sequencing and FMT technology and evaluated the criticality role of intestinal microbial homeostasis. Subsequently, metabolomics technologies, network pharmacology, and transcriptomics were combined were integrated to examine the anti-liver-fibrosis mechanisms and efficacy of SCU, providing a theoretical foundation and reference for the development and utilization of SCU as a drug against liver fibrosis.

## 2. Results

### 2.1. The Ameliorating Effect of SCU on Liver Fibrosis Rats Induced by CCl_4_

**Figure 1:**
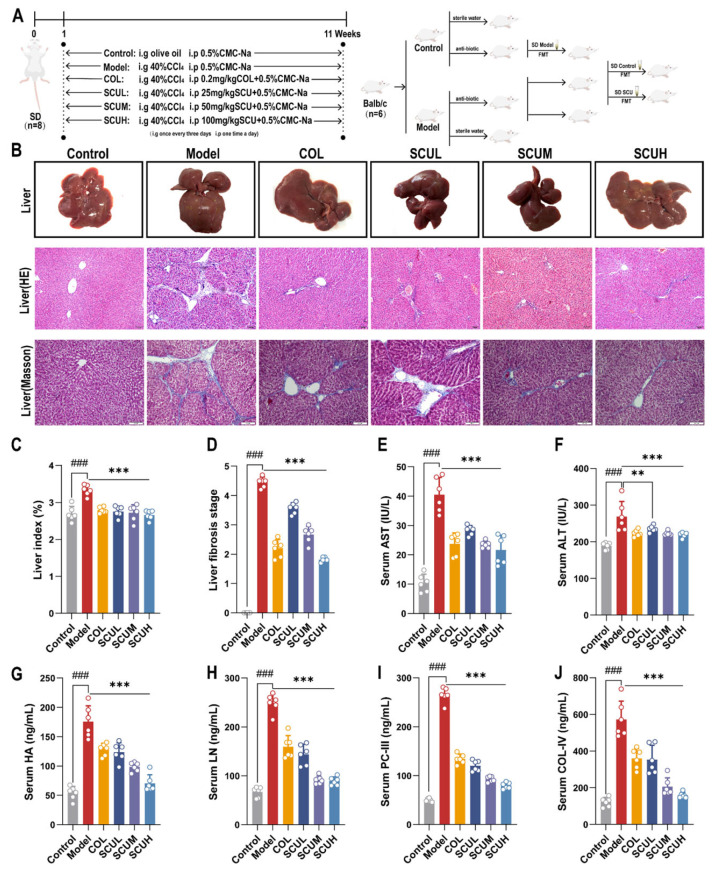
SCU reduced the degree of liver fibrosis induced by CCl_4_. (**A**) Schematic of animal experiment. (**B**) Representative histopathological images of rat liver (HE staining, 100×; Masson staining, 200×). (**C**) Liver index. (**D**) Liver fibrosis staging. (**E**–**J**) Serum AST, ALT, HA, LN, PC-III, and COL-IV. *n* = 6; data are presented as means ± standard deviations; ^###^ *p* < 0.001 vs. control group; ** *p* < 0.01, *** *p* < 0.001 vs. model group.

The effect of SCU was investigated on liver fibrosis rats established by CCl_4_ (Figure 1A). The administration of SCU significantly decreased the liver index induced by CCl_4_ (Figure 1C). Following repeated injections of CCl_4_, the serum concentrations of four indices of liver fibrosis, PC-III, COL-IV, HA, and LN, and liver injury indices, AST and ALT viability, were markedly increased, whereas the administration of SCU substantially reduced the serum levels of PC-III, COL-IV, HA, and LN alongside the dose-dependent efficacy of AST and ALT (Figure 1E–J).

Moreover, the liver morphology of the rats in the control group appeared normal. The livers of rats induced with the disease by CCl_4_ exhibited dark red coloration, swollen and rough, with yellow spots attached, accompanied with liver lobular adhesion. After SCU treatment, the livers displayed a ruddy and smooth appearance with reduced adhesions, resembling those of the control group. HE staining results showed that the normal rat liver lobule structure was unbroken and the hepatocytes were arranged normally; the central vein was radial without signs of inflammatory cell infiltration or fibroplasia. Conversely, the infiltration of the rat confluent area and central phlebitis cells induced by CCl_4_ was obvious, the structure of the liver lobules had been destroyed, and the fibrosis was serious. After the administration of SCU, there was a significant reduction in the degree of fibrous hyperplasia, a decrease in the number of lobular structural lesions within the liver, and an orderly arrangement of hepatocyte cords. Masson staining results indicated that CCl_4_ exposure led to extensive collagen fiber hyperplasia in the rat pipeline area and central vein, with pronounced fiber bridging observed. After SCU intervention, the degree of fibrosis was improved, and the fibrous tissue proliferation decreased. Notably, we found that the histological score was hugely reduced after giving SCU treatment, indicating a reduction in liver fibrosis (Figure 1B,D).

### 2.2. Effects of SCU on Intestinal Flora in CCl_4_-Induced-Liver-Fibrosis Rats

**Figure 2:**
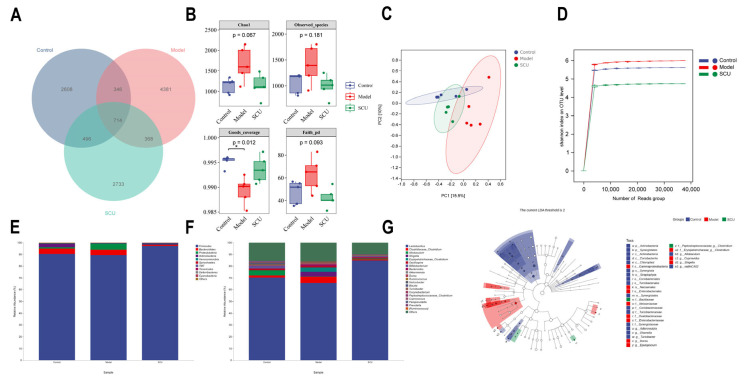
Microbiomes. (**A**) Analysis of intestinal flora in Venn diagram. (**B**) Analysis of intestinal flora diversity. (**C**) PCA. (**D**) Rarefaction curve. (**E**) Analysis of the phylum level. (**F**) Analysis of the genus level. (**G**) LEfSe analysis. *n* = 5. Data are presented as means ± standard deviations; control group; * *p* < 0.05 vs. model group.

The 16S rRNA gene from fecal samples was PCR-amplified, followed by high-throughput sequencing. The variety and complexity of the gut microbiota were evaluated using Chao1, observed species, and Goods coverage and Faith indexes (Figure 2A,B). Our findings indicate that the richness of intestinal flora increased after CCl_4_ induction while it recovered after SCU treatment. In the PCA plot, shorter axis distances reflect greater sample similarity, and a further distance indicates a lower sample similarity. After CCl_4_ induction, the flora structure differed significantly from control rats, and the flora structure in rats treated with SCU was closer to that in control rats. These results confirm that microbial flora undergoes significantly changes during liver fibrosis progression, and SCU plays a regulatory role in narrowing the difference between control rats and liver fibrosis rats (Figure 2C,D).

The phylum-level results indicated that the abundance of *Bacteroidetes*, *Proteobacteria,* and *Verrucomicrobia* increased significantly after the repeated injection of CCl_4_ while *Firmicutes* levels dropped markedly. Notably, the abundance of the above bacteria was markedly reversed after SCU treatment (Figure 2E). At the genus level, the findings indicated that the abundance of *Lactobacillus*, *Bifidobacterium*, *Allobaculum*, *Odoribacter,* and *Turicibacter* had decreased significantly after CCl_4_ induction and the abundance of *Clostridium* and *Shigella* had increased significantly. Following SCU administration, the abundances of *Lactobacillus*, *Bifidobacterium*, and *Allobaculum* had increased markedly while the abundances of *Clostridium* and *Shigella* had decreased remarkably (Figure 2F).

Linear discriminant analysis (LDA) Effect Size (LEfSe) analysis is utilized to determine the characteristics of differences between groups and find the key bacterial groups that differ. In this study, we detected the difference in the advantages of bacterial communities between the three groups. According to the threshold LDA score of >2.0 used, the higher of the LDA score is, the more significant it is in comparison, and conversely, a lower score indicates reduced significance. We used the LEfSe method to identify the differentially enriched representative bacterial communities among the control, model, and SCU groups (LDA > 2.0). The findings indicated that there was a significant enrichment of twenty-three bacteria in the control group, twenty bacteria were notably concentrated in model rats, and three bacteria showed marked enrichment in the SCU group. Among them, the main changes in the intestinal flora were in *Allobaculum*, *Shigella*, *Clostridium*, *Bifidobacterium*, *Helicobacter*, and *Turicibacter* (Figure 2G).

### 2.3. Fecal Microbiota Transplantation Verifies That Intestinal Flora Can Improve Liver Fibrosis in Mice

**Figure 3:**
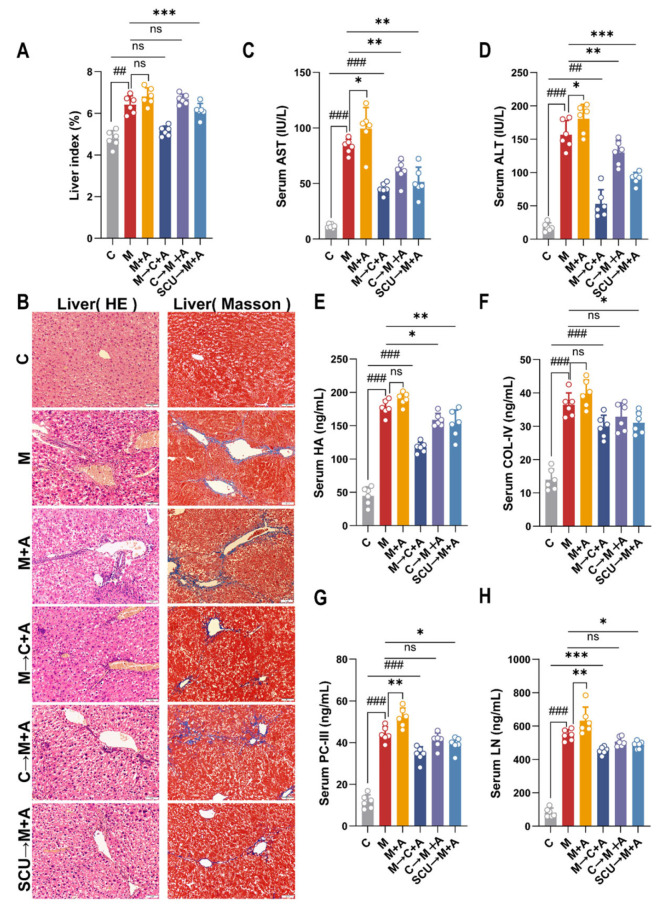
FMT verified that intestinal flora could reduce liver fibrosis in mice. (**A**) Liver index. (**B**) HE staining, 200×; Masson staining, 200×. (**C**–**H**) Serum AST, ALT, HA, LN, PC-III, and COL-IV. *n* = 6. Data are presented as means ± standard deviations; ^##^ *p* < 0.01, ^###^ *p* < 0.001 vs. control group; * *p* < 0.05, ** *p* < 0.01, *** *p* < 0.001 vs. model group. ns, *p* > 0.05.

The onset and advancement of liver fibrosis are strongly linked to the composition of the intestinal flora, and dysbiosis is often associated with the course of liver fibrosis [16,17]. Mice were administered with broad-spectrum antibiotics to establish a deplete bacterial flora state to examine the effects of changes in intestinal flora on liver fibrosis. Liver fibrosis mice that had been administered antibiotics to deplete the bacterial flora exhibited significant increases in the liver index, AST and ALT activity, and PC-III and LN contents (Figure 3A,C–H). In addition, liver fibrosis mice depleted of their bacterial flora were accompanied by hepatocyte necrosis with a massive infiltration of inflammatory cells, and a small quantity of blue collagen fiber growth was detected near the convergence zone and central vein without fiber bridging (Figure 3B). Interestingly, similar results to those of liver fibrosis mice depleted of bacterial flora were observed in model mice (M) and normal mice recolonized with model rat fecal bacteria (M→C + A).

The above indicators were significantly reduced in liver fibrosis mice recolonized with SCU rat fecal bacteria (SCU→M + A). Similarly, liver fibrosis mice recolonized with control rat fecal bacteria (C→M + A) showed significant reductions in other indicators except the liver index, COL-IV, and HA content (Figure 3A,C–H). In addition, the tissue morphology of untreated mice (C) was normal while the hepatocyte necrosis, inflammatory infiltration, and blue collagen fiber proliferation were significantly reduced in mice recolonized with fecal bacteria from the control or SCU groups, which was consistent with the pathological tissue results of rats directly administered with SCU (Figure 3B).

**Figure 4:**
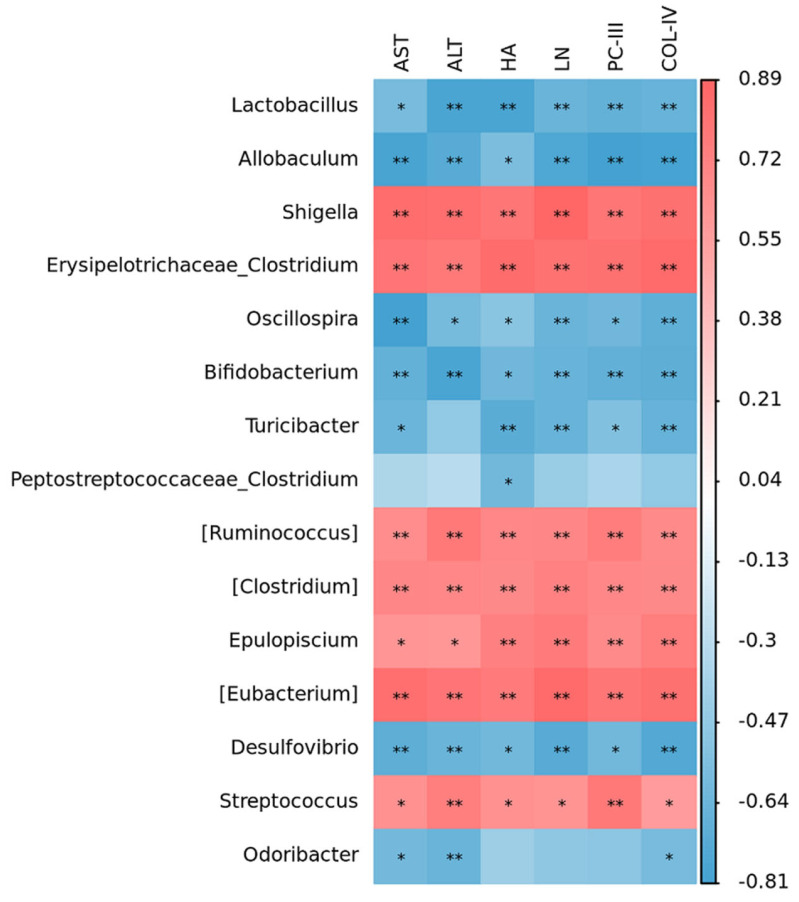
Spearman correlation analysis (intestinal flora vs. liver fibrosis). *n* = 3. Data are presented as means ± standard deviations; * *p* < 0.05, ** *p* < 0.01.

Spearman correlation analysis was carried out to evaluate the relationships among intestinal flora, metabolite, and liver fibrosis. It was found that *Lactobacillus*, *Allobaculum*, *Oscillospira*, *Bifidobacterium*, *Turicibacter*, *Desulfovibrio,* and *Odoribacte* exhibited remarkable negative correlations with AST, ALT, PCIII, COL-IV, HA, and LN (Figure 4).

### 2.4. Effect of SCU on Serum Metabolites in Liver Fibrosis Rats

**Figure 5:**
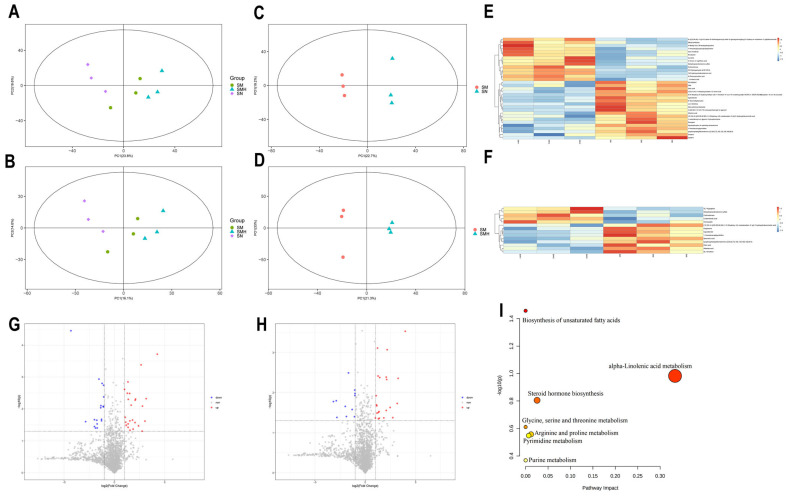
Metabolomics. (**A**) PCA in positive ion mode. (**B**) PCA in negative ion mode. (**C**) OPLS-DA in positive ion mode. (**D**) OPLS-DA in negative ion mode. (**E**) Heatmap of the number of DEGs in positive ion mode. (**F**) Heatmap of the number of DEGs in negative ion mode. (**G**) Volcano map analysis in positive ion mode. (**H**) Volcano map analysis in negative ion mode. (**I**) Results of serum differential metabolite KEGG enrichment. *n* = 3. Data are presented as means ± standard deviations.

The PCA of the unsupervised recognition mode showed that there was a distinct separation trend between the groups in both positive and negative ion modes (Figure 5A,B). Furthermore, the OPLS-DA analysis demonstrated that the serum samples exhibited excellent stability and the inter-group sample could be distinguished well (Figure 5C,D). Heatmaps were used to determine the differences in the metabolite spectra of the serum samples between groups, and the metabolic phenotypes of the differences changed remarkably (Figure 5E,F). The variable weight value (VIP) obtained according to the OPLS-DA model could effectively estimate the groups and show a clear trend of being separated in the context of positive and negative ions. This approach facilitated the identification of biologically profound metabolites. VIP > 1 and *p* < 0.05 were used as screening standards to distinguish the significant differences in metabolites across groups (Figure 5G,H). A total of seven metabolites demonstrated crucial changes in serum samples, as presented in Table 1.

To further elucidate the metabolic pathways that may influence liver fibrosis, MetaboAnalyst software 5.0 was used to enrich and analyze the metabolic pathways associated with these metabolites. In serum samples, the distinct metabolites were mainly involved in the following seven metabolic pathways: the synthesis of unsaturated fatty acids, the metabolic pathways of α-linolenic acid, the production of steroid hormones, and the metabolic processes involving glycine, serine, and threonine, alongside the metabolism of arginine and proline, and the pathways for pyrimidine and purine metabolism (Figure 5I).

### 2.5. Correlation Within Metabolite–Liver Fibrosis–Intestinal Flora

**Figure 6:**
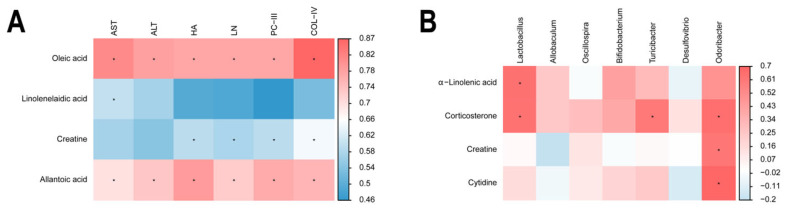
Spearman correlation analysis. (**A**) Metabolites vs. liver fibrosis. (**B**) Metabolites vs. intestinal flora. *n* = 3. Data are presented as means ± standard deviations; * *p* < 0.05.

Subsequently, we carried out an investigation on the correlations between metabolites and the findings demonstrated that the liver fibrosis index had a positive correlation with oleic acid and allantoic acid whereas it had a negative correlation with Linolenelaidic acid and creatine (Figure 6A). Furthermore, the analysis indicated that the intestinal flora *Lactobacillus*, *Turicibacter,* and *Odoribacter* had positive correlations with corticosterone, creatine, and cytidine (Figure 6B).

### 2.6. Network Pharmacological Analysis of SCU for the Treatment of Liver Fibrosis

**Figure 7:**
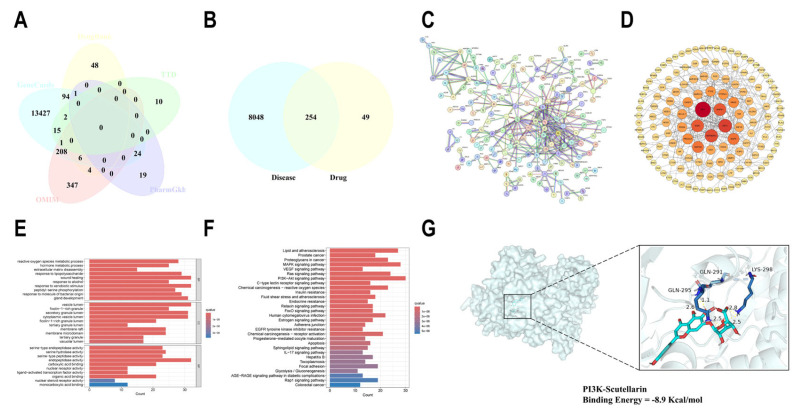
Network pharmacological analysis. (**A**) Nonrepetitive target Venn diagram of liver fibrosis. (**B**) The target Venn diagram depicting the intersection of SCU and liver fibrosis. (**C**) SCU and liver fibrosis intersection target PPI network. (**D**) Liver fibrosis target PPI diagram. (**E**) GO enrichment analysis of the intersection target of SCU and liver fibrosis. (**F**) KEGG analysis of the intersection target of SCU and liver fibrosis. (**G**) Molecular docking results of PI3K and SCU.

We obtained a total of 303 SCU targets with official symbols and 8302 nonrepetitive targets closely related to liver fibrosis (Figure 7A) with a total of 254 intersecting targets between the two datasets (Figure 7B). In the PPI network graph, nodes that were darker and larger exhibited greater importance (Figure 7C,D). PPI analysis revealed strong correlations between diseases, drugs, and the highly cored genes. GO enrichment analysis obtained 1989 entries for biological processes (BPs), 72 entries for cell composition (CC), and 190 items for molecular function (MF) (Figure 7E). The results of MF analysis indicated that SCU may exert its effects by affecting endopeptidase activity and serine hydrolase activity. The findings from CC analysis showed that SCU mainly influenced the vesicle lumen and secretory granule lumen. Furthermore, the BP analysis demonstrated that SCU may exert its effects through wound healing and response to exogenous stimuli. At the same time, 186 related pathways were obtained through KEGG pathway enrichment analysis (Figure 7F). Among them, SCU mainly acts on signaling pathways such as PI3K/AKT, which mainly exert regulatory effects by modulating inflammation and lipid metabolism. The molecular docking binding energy between PI3K and SCU was −8.9 Kcal/mol, indicating that they had good binding ability (Figure 7G).

### 2.7. Effects of SCU on Transcriptomics in CCl_4_-Induced-Liver-Fibrosis Rats

**Figure 8:**
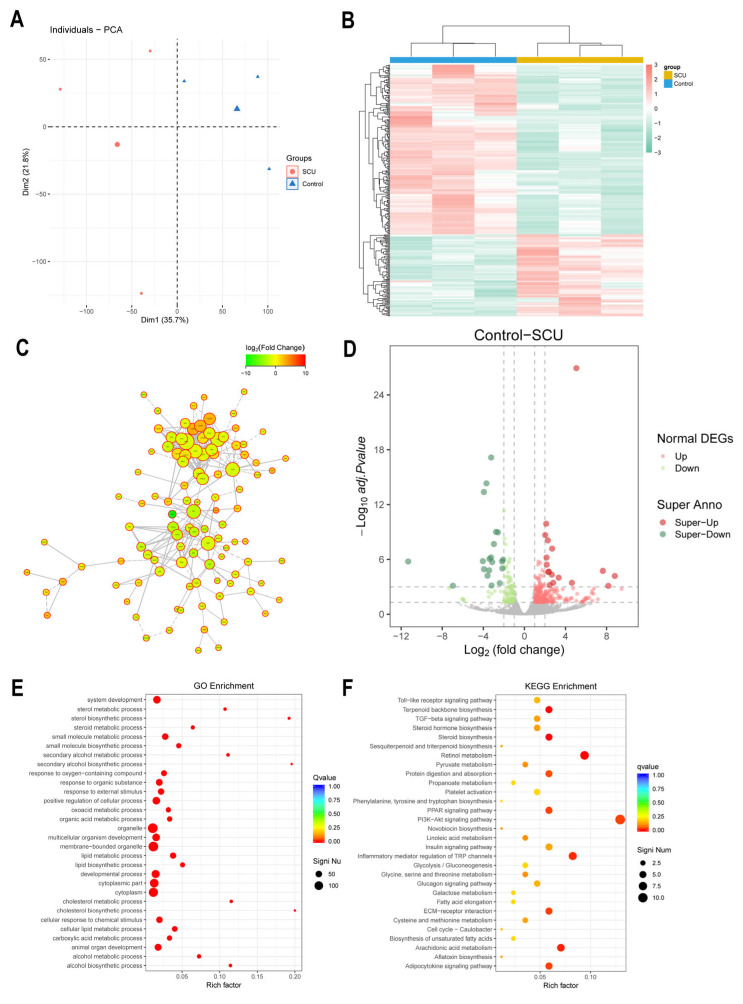
Transcriptomics. (**A**) PCA. (**B**) DEG hierarchical clustering analysis. (**C**) PPI network. (**D**) Volcano map. (**E**) GO. (**F**) KEGG.

We analyzed the sequencing of the liver transcriptome to explore the potential molecular mechanism of SCU to improve liver fibrosis. In order to identify vital difference genes, the screening thresholds were set to a q value < 0.05 and the fold change difference with an absolute value |Fold Change| > 2. PCA (Principal Component Analysis) showed some separation between the groups (Figure 8A). A heatmap illustrating differential gene expression between groups is shown in Figure 8B. The PPI network highlighted correlations among differentially expressed genes (DEGs) (Figure 8C). The results of differential gene screening showed that the expression of 187 genes including Taok1, Acta1, Eln, Cyp4a10, and Cyp2a4 in the samples underwent dynamic changes (Figure 8D).

The biological processes in which these genes are involved are mainly associated with metabolic processes and immune system processes. The entries related to cellular components predominantly focus on organelles, extracellular matrix components, etc. In terms of molecular function, the entries mainly involve catalytic activity, molecular function regulators, and antioxidants. The GO functional enrichment results showed that the genes with dynamic changes were mainly enriched in cell apoptosis and proliferation, inflammatory response, oxidative stress, and other aspects (Figure 8E). KEGG enrichment analysis indicated that the genes with identified expression were clustered in signaling pathways relevant to lipid metabolism, amino acid biosynthesis, inflammation, oxidative stress, and cell proliferation (Figure 8F). Notably, the PI3K/AKT axis contained the most enriched genes, which aligned with the outcomes of network pharmacology.

**Figure 9:**
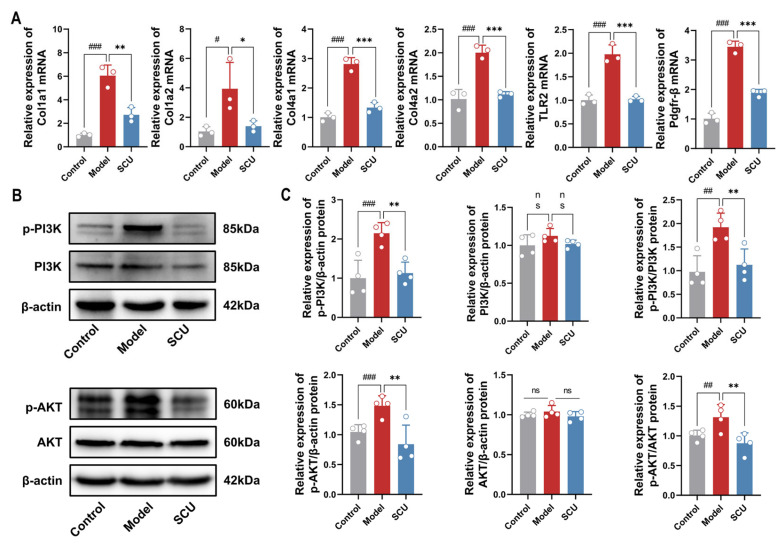
The expression of genes and proteins related to thePI3K/AKT signaling pathway. (**A**) Relative expression levels of mRNA *Col1a1*, *Col1a2*, *Col4a1*, *Col4a2*, *TLR2*, and *Pdgfr-β*. (**B**) Expression of proteins p-PI3K, PI3K, p-AKT, AKT, and β-actin. (**C**) Relative expression levels of p-PI3K/β-actin, PI3K/β-actin, p-PI3K/PI3K, p-AKT/β-actin, AKT/β-actin, and p-AKT/AKT; *n* = 4. Data are presented as means ± standard deviations; ^#^ *p*< 0.05, ^##^ *p* < 0.01, ^###^ *p* < 0.001 vs. control group; * *p* < 0.05, ** *p* < 0.01, *** *p* < 0.001 vs. model group. ns, *p* > 0.05.

On the basis of transcriptomics and network pharmacology findings, genes connected with liver fibrosis and the PI3K/AKT signaling pathway, such as *Col1a1*, *Col1a2*, *Col4a1*, *Col4a2*, *TLR2*, and *Pdgfr-β*, were chosen for qRT-PCR validation. SCU markedly decreased the expression of these genes (Figure 9A). The protein levels of the PI3K/AKT signaling pathway were further verified by WB (Figure 9B). As depicted in Figure 9C, SCU obviously reduced the protein expression of p-PI3K and p-AKT in comparison to the model group. The combined outcomes from transcriptomics, qRT-PCR, and WB indicated that SCU significantly suppressed the PI3K/AKT signaling pathway and its associated genes in the CCl_4_-induced rat model.

## 3. Discussion

Liver fibrosis is an aberrant reparative process triggered by various chronic liver damages. Hepatic stellate cells (HSCs) are activated when liver cells are damaged. HSCs transdifferentiate into myofibroblast-like cells, secreting extracellular matrix (ECM) components (such as collagen) and profibrotic factors. This further promotes cell damage, thus inducing fibrosis [18]. Scutellarin (SCU) is a flavonoid component with antioxidant, anti-inflammatory, and hepatoprotective effects. Although previous studies have demonstrated the protective effects of SCU against liver injury and fibrosis, its underlying anti-liver-fibrosis mechanisms are still unclear [6,7]. In the course of this research, the pharmacodynamic impact of SCU on liver fibrosis was demonstrated using the CCl_4_-induced liver fibrosis model. Moreover, studies found that SCU treatment improved the repair of intestinal microbial dysbiosis in liver fibrosis rats and enhanced the ratio of the abundance of *Firmicutes* to that of *Proteobacteria*. In addition, it also enhanced the abundance of *Lactobacillus*, *Bifidobacterium,* and *Allobaculum* and decreased the abundance of *Clostridium* and *Shigella*. Most of the Firmicutes members are probiotics capable of generating short-chain fatty acids (SCFAs) and fortify the intestinal barrier [10]. Among them, *Lactobacillus*, *Allobaculum*, and *Turicibacter* regulate gut microbiota balance by adjusting the expression of endogenous antibacterial substances in the intestine, reduce the proliferation of harmful bacteria, and inhibit inflammatory and fibrosis processes, which is consistent with the function of *Bifidobacterium* in *Actinobacteria* [19,20]. *Clostridium* mediates 7α-dehydroxylation activity, which activates the biosynthesis of secondary bile acids, thereby inhibiting liver Farnesoid × receptor signaling and affecting liver metabolism and inflammation. In addition, it accelerates the advancement of hepatocellular carcinoma by influencing the intestinal barrier functionality and immune response. *Odoribacter* is a common SCFA-producing microorganism, and its deficiency is associated with many metabolic and immune diseases. *Odoribacter splanchnicus* can secrete outer membrane vesicles in the intestinal epithelium and exert anti-inflammatory effects [21]. *Proteobacteria* have active endotoxins, among which members of the *Enterobacteriaceae* family can penetrate the damaged intestinal barrier and stimulate the innate immune system, causing the chronic inflammation of the intestinal wall and liver [10].

Broad-spectrum antibiotics were used to clear the recipient’s intestinal microbiota. This reduction in bacterial load facilitated more effective colonization by donor microorganisms, and different antibiotic combinations could achieve the maximum effect of clearing intestinal flora [22]. Our study found that antibiotic administration and receiving model group feces led to more severe liver-fibrosis-related disease phenotypes, suggesting that the intestinal flora are implicated in the development of CCl_4_-triggered liver fibrosis. In addition, we observed that transplanting SCU-treated fecal microbiota into mice with liver fibrosis significantly reversed the progression of liver fibrosis, further confirming that SCU can modulate dysbiosis and that transplanting this stable intestinal flora can ameliorate liver fibrosis.

Notably, SCU can regulate the levels of metabolites such as linoleic acid, oleic acid, corticosterone, creatine, allantoic acid, and cytidine. The Spearman correlation analysis revealed that the liver fibrosis index showed a positive association with oleic acid and allantoic acid while displaying an inverse relationship with linoleic acid and creatine. Research has shown that the intestinal flora can metabolize lipids and generate bioactive metabolites [23]. It has been reported that alterations in the levels of linoleic acid are related to the development of liver fibrosis [24]. Research has shown that intestinal flora (such as *Lactobacillus* and *Bifidobacterium*) can transform linoleic acid into conjugated linoleic acid via linoleic acid isomerase. Conjugated linoleic acid has the function of resisting infection and regulating inflammation [25,26]. Additionally, research indicates that the supplementation of creatine can reduce liver fibrosis, inflammation, and the oxidative stress response caused by doxorubicin [27]. Corticosterone administration can alleviate liver inflammation and fibrosis by activating the Hypothalamic–Pituitary–Adrenal axis [28]. Uric acid may become a factor promoting liver fibrosis by directly inducing liver fat deposition and oxidative stress, increasing the level of inflammatory factor, etc. In the intestine, intestinal flora, such as *Lactobacillus* and *Bifidobacterium*, convert uric acid into allantoic acid and ultimately urea by secreting specific enzymes (such as uricase, allantoinase, and allantocystase). At the same time, they can secrete metabolites such as SCFAs to promote the elimination of allantoic acid, thereby reducing uric acid in the body [29,30]. Increased oleic acid levels are associated with enhanced triglyceride synthesis in the liver, triggering liver disease [31]. In addition, Spearman correlation analysis found that intestinal flora such as *Lactobacillus*, *Turicibacter,* and *Odoribacter* were positively correlated with corticosterone, creatine, and cytidine. They may play anti-liver-fibrosis roles by secreting specific enzymes to affect metabolites.

We employed network pharmacology and transcriptomics to further explore the specific molecular pathways involved. KEGG enrichment analysis revealed that differentially expressed genes were mainly linked to lipid metabolism pathways, oxidative stress, and inflammation, among which the PI3K/AKT signaling pathway had the most highly enriched genes. Molecular docking also showed that SCU and PI3K could stably bind. The PI3K/AKT/mTOR signaling pathway promotes liver fibrosis progression through cellular growth regulation, differentiation, and programmed cell death and promoting epithelial-mesenchymal transition (EMT) [32,33]. In addition, the PI3K/AKT signaling pathway is critical for regulating macrophage survival, migration, and proliferation and its response to metabolic and inflammatory signals [34]. This pathway is as a possible target for chronic liver disease treatment, including for the treatment of liver fibrosis [18]. Among the genes, further analysis disclosed that the gene expression levels of *TLR2*, *Col1a1*, *Col1a2*, *Col4a1*, *Col4a2*, and *Pdgfr-β* were changed during the SCU anti-liver-fibrosis process, and qRT-PCR verified this result. Toll-like receptor 2 (*TLR2*) participates in the activation of liver immune cells, stellate cells, and the PI3K/AKT signaling pathway and performs a particular function in regulating inflammation and liver fibrosis [35,36]. *Col1a1* and *Col1a2* are genes encoding the collagen Iα chain that are involved in the accumulation of ECM. They increase in activated HSCs and regulate the advancement of liver fibrosis as indicators of liver fibrosis and possible treatment objectives. The body reduces liver fibrosis through downregulating the expression of *Col1a1* and *Col1a2*, thereby affecting the PI3K/AKT signaling pathway to induce HSC senescence [37,38]. Platelet-derived growth factor receptor (*Pdgfr*) is recognized as the strongest mitogen for activated HSCs [39]. *Col4a2* and *Pdgfr-β* are liver-fibrosis-specific genes associated with the PI3K/AKT signaling pathway, and their expression levels are intimately tied to the severity of liver fibrosis [40]. This indicates that the PI3K/AKT signaling pathway is crucial in the anti-liver-fibrosis process of SCU.

Moreover, intestinal flora disturbance causes the release of inflammatory factors, thereby triggering the PI3K/AKT/mTOR signaling pathway [41]. Our previous findings showed that SCU significantly decreased inflammatory factor levels in liver-injury-model mice [6]. This suggests that SCU may inhibit the PI3K/AKT pathway by regulating intestinal flora to inhibit the release of inflammatory factors. However, its precise action mechanism still needs to be further explored through subsequent experiments.

## 4. Materials and Methods

### 4.1. Materials and Reagents

We used SCU (purity > 98%, HB201212-01) and Colchicine (20201105), purchased from Yunnan Herbal Medicine Industry Co., Ltd. (Kunming, China); CCl4 (purity > 99.5%, 20190706, Tianjin Fuchen Chemical Reagent Factory, Tianjin, China); Carboxymethylcellulose Sodium (20190604, China National Pharmaceutical Group Chemical Reagent Co., Ltd., Shanghai, China); Isoflurane (64201201, Lunan Better Pharmaceutical Co., Ltd., Linyi, China); Glutamate Oxaloacetate Transaminase (AST, 20211224) and Glutamate Pyruvate Transaminase (ALT, 20211223) Test Kits, purchased from Nanjing Jiancheng Bioengineering Institute (Nanjing, China); hyaluronic acid (HA, 20211220), layer adhesion protein (LN, 20211214), type III pre-collagen (PC-III, 20211220), and type IV collagen (COL-IV, 20211220), purchased from Shanghai Enzyme-Linked Biotechnology Co., Ltd. (Shanghai, China); Total RNA Extractor (Trizol, B5-11311, Shenggong Bioengineering (Shanghai) Co., Ltd., Shanghai, China); RNA extraction kit (dp451), FastKing RT Kit With gDNase (KR116-02), SuperReal PreMix Plus SYBR Green (FP205-02), purchased from Tiangen Biotech (Beijing) Co., Ltd. (Beijing, China); RIPA buffer (R0010), PMSF (P0100), protein phosphatase inhibitor (P1260), and SDS-PAGE (P1200), purchased from Beijing Solarbio Science & Technology Co., Ltd. (Beijing, China); PVDF membrane (Merck Millipore, IPVH00010, Burlington, MA, USA); QuickBlock™ Western Blocking solution (Beyotime, P0252, Shanghai, China); goat Anti-Rabbit IgG (Proteintech, SA00001-2, Wuhan, China); and ECL chemiluminescence substrate (Biosharp, BL520B, Hefei, China).

### 4.2. Animal Study

Male Sprague Dawley (SD) rats, weighing 180–220 g and of SPF grade, were used. The animal study received approval from Dali University’s Animal Ethics Committee (Approval No. 2021-P2-82). Rats were separated into 6 groups in a random manner: control group, model group, Colchicine group (COL: 0.2 mg/kg), and scutellarin group (SCUL: 25 mg/kg; SCUM: 50 mg/kg; SCUH: 100 mg/kg). The rats in the control group were administered 1 mL/kg of olive oil via peritoneal injection every 3 days while the remaining rats received an equivalent volume of 40% CCl_4_ solution for 10 weeks. Concurrently, the stomach was treated with a drug dose of 10 mL/kg. Both the control and model groups were provided with an equal amount of 0.5% CMC-Na solution once a day for 10 weeks. The dosage of the drug was determined according to the Chinese Pharmacopoeia and the results of previous studies.

Fecal microflora transplantation (FMT): Male BALB/c mice, weighing 18–22 g and of SPF grade, were used. A total of 36 BALB/c mice were assigned randomly to the control group (C, *n* = 12) and model group (M, *n* = 24). Following the successful establishment of the model, 6 mice from the C group and 18 mice from the M group were randomly selected to receive gastric pretreatment with the antibiotic mixture (1 g/L ampinicillin, 0.5 g/L vancomycin, 1 g/L neomycin sulfate, 1 g/L metronidazole, 200 μL/day), for an interval of 12 h, for constructing control group with depleted intestinal flora (C + A) and model group with depleted intestinal flora (M + A) [42]. Antibiotics had to be discontinued at least 12 h before FMT. Fresh feces samples were collected from each group of rats, and the mice (6 per group) underwent FMT as follows: (1) C group, M group and M + A group: we gave the rats an equal amount of sterile water by tube feeding every day; (2) M→C + A group: model group fecal bacteria were transplanted to C + A group; (3) C→M + A: control group fecal bacteria were transplanted to M + A group; and (4) SCU→M + A: SCU group fecal bacteria were transplanted to the M + A group. The administration was given for 3 consecutive days, followed by every other day, a total of 6 times.

Each animal was fasted for 12 h following the last administration of the drug, after which it was executed for follow-up experiments. The detailed schematic of the animal experiment design is presented in Figure 1A.

### 4.3. Biochemical Assays

The biochemical kit was used to determine the levels of aspartate transaminase (AST) and alanine transaminase (ALT), which served as indicators of liver damage in the serum sample. The ELISA kit was employed to detect the content of the four indicators of liver fibrosis in the serum sample: hyaluronic acid (HA), layer adhesion protein (LN), type III pre-collagen (PC-III), and type IV collagen (COL-IV) [43,44].

### 4.4. Histological Analysis

The liver tissue was fixed in 4% paraformaldehyde and subsequently buried in paraffin. H&E (Haematoxylin and Eosin) and Masson staining were used for pathological evaluation. Masson staining specifically assesses the degree of fibrosis. We took slice photos under the microscope (OLYMPUS, Tokyo, Japan) and evaluated the degree of liver fibrosis according to the Ishak scoring system [45].

### 4.5. 16S rRNA Sequencing

Total DNA was extracted from the stool specimens of rats. PCR amplification, electrophoresis, magnetic beads on the bacterium 16S rRNA gene V3–V4 region using forward primers 5′-ACTCCTACGGGAGGCAGCA-3′ and reverse 5′-GGACTACHVGGGTWTCTAAT-3′, electrophoresis separation, magnetic bead purification, and recycling were performed. The product was fluorescence-quantified, after which the sample was mixed in the corresponding proportion according to the fluorescence quantification results and the sequencing requirements of the sample. The sequencing platform was the MiSeq platform of llumina, which analyzes bioinformatics methods according to the QIIME2 analysis process [46,47].

### 4.6. Serum Metabolomics Analysis

#### 4.6.1. Sample Preparation and Quality Control

We ground the serum sample and then added 400 μL pre-cooled methanol/acetonitrile/aqueous solution (4:4:2, *v*/*v*); performed vortex mixing, −20 °C; after 1 h, performed 14,000× *g* 4 °C centrifuging for 20 min; took supernatant; vacuum-dried it; conducted mass spectrometry analysis; added 100 μL aqueous solution (acetonitrile:water = 1:1, *v*/*v*) to dissolve and vortex it; performed 14,000× *g* 4 °C centrifuging for 15 min; and took 2 μL supernatant for analysis.

To evaluate the stability of the system and the credibility of experimental data, a uniformly mixed sample (QC sample) consisting of all samples was placed into the sample sequence following each group of samples.

#### 4.6.2. Instrumental Analysis Methods

Chromatographic separation of all samples was processed using an ACQUITY UPLC BEH C18 column (100 mm × 2.1 mm, 1.7 μm, Waters, Milford, MA, USA). The samples were detected by electrospray ionization (ESI) and operated in both positive and negative ion modes.

### 4.7. Metabolite Identification and Pathway Analysis

The raw data collected by mass spectrometry were preprocessed through peak extraction, peak alignment, peak adjustment, and standardization. Pattern recognition analysis was carried out using SIMCA-P 14.1 software and the data were preprocessed by Pareto-scaling for multivariate statistical analysis. The structure of each metabolite was identified according to the technique of accurate mass number matching (<25 ppm) and secondary spectral correlation, and the metabolite was searched and compared through the laboratory’s self-built database and Biocyc, HMDB, liver fibrosis MDB, Lipidmaps, and other databases to clarify the metabolites name and speculate on the metabolic pathway.

### 4.8. Network Pharmacology and Molecular Docking

The TCMSP (https://old.tcmsp-e.com/tcmsp.php), Swiss Target Prediction (http://www.swisstargetprediction.ch/), SEA (https://sea.bkslab.org/), and Pharmapper databases (http://www.lilab-ecust.cn/pharmmapper/) were used to predict the potential target information of SCU. We collected disease-related targets for “hepatic fibrosis” or “liver fibrosis” from the GeneCards (https://www.genecards.org/), OMIM (https://www.genecards.org/), DisGeNet (https://disgenet.com/), PharmGkb (https://www.pharmgkb.org/), and DrugBank databases (https://www.drugbank.com/). Subsequently, we determined the intersection of disease targets and SCU targets. The intersection target was imported into the STRING database (https://cn.string-db.org/); we restricted the species to “human”, set the protein interaction confidence score to 0.900, excluded a single unrelated node, and obtained protein interaction information. Cytoscape 3.9.0 software was used to import these data to build protein–protein interaction (PPI) networks, using the CytologyNCA plug to construct a PPI network between the target and the drug for topological analysis. The sizes and color depths of the nodes signified the magnitude of their respective values. The “BiocManager” package of R 4.3.1 language software was used to perform Gene Ontology (GO) functional enrichment analysis and Kyoto Encyclopedia of Genes and Genomes (KEGG) pathway enrichment analysis to screen key biological functions and signaling pathways (*p* value < 0.05). The structures of SCU and target proteins were downloaded from PubChem database (https://pubchem.ncbi.nlm.nih.gov/) and PDB database (https://www.pdbus.org/), all accessed on 27 March 2024. AutoDockTools 1.5.7 software was used to hydrogenate and format the target protein molecules and obtain their active pockets. Molecular docking was carried out by VINA 1.1.2 software and then visualized using Pymol 2.5.4 software.

### 4.9. Transcriptome Sequencing of Liver Tissue

TRIzol^®^ reagent was used to extract total RNA from liver tissue. The quality and purity of the total RNA were evaluated using the Qubit RNA Assay Kit. PE libraries were prepared; mRNA was purified, fragmented, and served as templates; and the synthesis of first- and second-strand cDNA was carried out with specific primers and reverse transcriptase. The generated double-stranded cDNA fragments were linked to adapter sequences and amplified via PCR. PCR products were purified and the quality of the library was assessed. The sequencing was carried out on the Illumina HiSeq platform. The generated data were applied for bioinformatics analysis. FastQC V0.11.9 software was employed to evaluate the quality of the raw sequences to achieve clear readings for further analysis. DESeq was employed for analyzing differential gene expression across groups.

### 4.10. qRT-PCR Analysis

RNA extraction kit was utilized to isolate RNA from rat liver tissue. cDNA was synthesized using FastKing RT Kit with gDNase according to the standard procedure. SuperReal PreMix Plus SYBR Green was used for quantitative PCR analysis on a real-time fluorescence quantitative PCR instrument.

The primers sequences are shown in Table 2. The relative expression of the target gene mRNA was calculated by 2^−ΔΔCt^ method and visualized with GraphPad Prism 9.5.

### 4.11. Western Blotting

Rat liver tissues were homogenized in RIPA buffer containing PMSF and phosphatase inhibitor. The denatured protein was quantified to a concentration of 30–40 μg and subjected to electrophoresis on an SDS-PAGE gel, then subsequently transferred to the PVDF membrane. To block the membrane, QuickBlock™ Western Blocking solution was applied at 25 °C for 20 min and then incubated overnight at 4 °C with primary antibodies. Membranes were then treated with either second antibody at 25 °C for 1 h. The proportions and dilutions of antibodies are shown in Table 3. Enhanced chemiluminescence was utilized to visualize the protein bands; quantitative analysis was performed using Image J software 1.8.0.

### 4.12. Statistical Analysis

All data were shown means ± standard deviations. SPSS 26.0 software was utilized to conduct a homogeneity test of variance and one-way ANOVA to compare multiple groups of data to assess statistical significance. *p* < 0.05 was considered as statistically significant.

## 5. Conclusions

To conclude, SCU may ameliorate liver fibrosis by altering the abundance of *Bifidobacterium*, *Lactobacillus*, and *Allobaculum*; regulating linoleic acid, the glycerophospholipid metabolism, and the production of unsaturated fatty acids; and downregulating PI3K/AKT signaling pathway. Multi-omics revealed that the effects of SCU on liver fibrosis are multi-target and multi-level; whether SCU mediates its anti-liver-fibrosis effect by regulating the intestinal flora–metabolite network to inhibit the PI3K/AKT pathway remains to be further explored. This research provides a new perspective for identifying therapeutic targets and effective pharmacological interventions for liver fibrosis and establishes a scientific basis for research on the development of new drugs and clinical applications of SCU.

## Figures and Tables

**Table 1:** Serum differential metabolites (*n* = 3).

Metabolites	Formula	RT	Model vs. Control			SCU vs. Model		
			Fold Change	Trend	VIP	Fold Change	Trend	VIP
Oleic acid	C_18_H_34_O_2_	9.00	1.15	Up ^#^	1.34	0.82	Down *	1.36
Linolenelaidic acid	C_18_H_30_O_2_	8.96	0.72	Down ^#^	1.68	1.38	Up *	1.23
Dehydroepiandrosterone sulfate	C_19_H_28_O_5_S	10.78	0.78	Down ^##^	1.19	1.48	Up **	1.97
Corticosterone	C_21_H_30_O_4_	6.65	0.14	Down ^##^	1.41	2.25	Up **	1.76
Creatine	C_4_H_9_N_3_O_2_	1.11	1.01	Up ^#^	1.19	0.72	Down *	1.72
Cytidine	C_9_H_13_N_3_O_5_	1.14	0.89	Down ^#^	1.57	0.6	Down *	1.76
Allantoic acid	C_4_H_8_N_4_O_4_	0.99	1.29	Up ^#^	1.24	0.53	Down *	1.91

^#^ *p*< 0.05, ^##^ *p* < 0.01 vs. control group; * *p* < 0.05, ** *p* < 0.01 vs. model group.

**Table 2:** PCR primers.

Gene		Primer (5′ to 3′)	Product Length
*Col1a1*	FP	TGTTGGTCCTGCTGGCAAGAATG	5843 bp
	RP	GTCACCTTGTTCGCCTGTCTCAC	
*Col1a2*	FP	GGGCAACAGCAGATTCACCTACAC	4465 bp
	RP	CAAGGAATGGCAGGCGAGATGG	
*Col4a1*	FP	ACAGCCAGGGATGCCAGGAAG	6579 bp
	RP	CACGACTACCAGGAAAGCCAACTC	
*Col4a2*	FP	GGGACCTGCCATTACTTCGCTAAC	6382 bp
	RP	GGATGGTGTGCTCTGGAAGTTCTG	
*TLR2*	FP	TCTGGAGTCTGCTGTGCCCTTC	2614 bp
	RP	GGAGCCACGCCCACATCATTC	
*Pdgfr-β*	FP	CTTGTTCTGGGACGCACTCTTGG	5405 bp
	RP	GCTTCTCACTGCTTCTGGCTGTAG	

**Table 3:** Primary antibodies for Western blotting.

Antibody	Host	Clonality	Dilution	Manufacturer	Cat. No.
Phospho-PI3K (Tyr607)	Rabbit	Polyclonal	1:1000	Abcam, Waltham, MA, USA	ab182651
PI3K	Rabbit	Polyclonal	1:1000	Wanleibio, Shenyang, China	WL02240
Phospho-AKT (Ser473)	Rabbit	Polyclonal	1:1000	Wanleibio, Shenyang, China	WLP001a
AKT	Rabbit	Polyclonal	1:1000	Zenbio, Chengdu, China	342529
β-actin	Rabbit	Monoclonal	1:1000	Cell Signaling Technology, Danvers, MA, USA	4970

## Data Availability

The original data presented in the study are openly available in Science Date Bank at https://doi.org/10.57760/sciencedb.18621.

## Abbreviations

The following abbreviations have been used in this manuscript:

AKT (PKB)	Protein Kinase B
ALT	Alanine aminotransferase
AST	Aspartate aminotransferase
BP	Biological process
CC	Cell composition
CCl4	Carbon tetrachloride
COL	Colchicine
*Col1a1*	Collagen Type I Alpha 1
*Col1a2*	Collagen Type I Alpha 2
*Col4a1*	Collagen Type IV Alpha 1
*Col4a2*	Collagen Type IV Alpha 2
COL-IV	Type IV collagen
DEGs	Differentially expressed genes
ECM	Extracellular matrix
FMT	Fecal microflora transplantation
GO	Gene Ontology
H&E	Haematoxylin and Eosin
HA	Hyaluronic acid
HSCs	Hepatic stellate cells
KEGG	Kyoto Encyclopedia of Gene and Genome
LDA	Linear discriminant analysis
LEfSe	Linear discriminant analysis Effect Size
LN	Layer adhesion protein
MF	Molecular function
mTOR	Mechanistic Target of Rapamycin
OPLS-DA	Orthogonal Partial Least Squares Discriminant Analysis
PCA	Principal Component Analysis
PC-III	Type III pre-collagen
*Pdgfr-β*	Platelet-derived growth factor receptors-β
PI3K	Phosphatidylinositol 3-kinase
PPI	Protein–protein interaction
qRT-PCR	Quantitative reverse-transcription polymerase chain reaction
SCFAs	Short-chain fatty acids
SCU	Scutellarin
*TLR2*	Toll-like receptor 2
VIP	Variable importance in the projection
